# Challenges of helicopter mountain rescue missions by human external cargo: need for physicians onsite and comprehensive training

**DOI:** 10.1186/s13049-019-0598-2

**Published:** 2019-02-13

**Authors:** Urs Pietsch, Giacomo Strapazzon, Dimitri Ambühl, Volker Lischke, Simon Rauch, Jürgen Knapp

**Affiliations:** 1 Department of Anaesthesiology and Intensive Care Medicine, Cantonal Hospital St. Gallen, Rorschacher Strasse 95, 9007 St. Gallen, Switzerland; 2 Air Zermatt, Emergency Medical Service, 3920 Zermatt, Switzerland; 3 Bergwacht Schwarzwald, Hessen, Bayern Germany; 4 Institute of Mountain Emergency Medicine, Eurac Research, Bozen, Italy; 5 CNSAS Italian Mountain Rescue, Milan, Italy; 6 Medical School, University of Bern, Bern, Switzerland; 7 Department of Anaesthesiology and Pain Medicine, Inselspital, Bern University Hospital, University of Bern, 3010 Bern, Switzerland; 8 Department of Anaesthesiology, University Hospital of Munich (LMU), Munich, Germany

**Keywords:** Trauma, Helicopter emergency medical service, Training, Emergency medicine, Alpine rescue

## Abstract

**Background:**

Human external cargo (HEC) extrication during helicopter rescue missions is commonly used in mountain emergency medical services. Furthermore, longline or winch operations offer the opportunity to deliver professional medical care onsite. As the safety and quality of emergency medical care depends on training and experience, we aimed to investigate characteristics of mountain rescue missions with HEC.

**Methods:**

We retrospectively reviewed all rescue missions conducted by Air Zermatt (a commercial rescue service in the high-alpine region of Switzerland) from January 2010 to September 2016.

**Results:**

Out of 11,078 rescue missions 1137 (10%) required a HEC rescue. In 3% (*n* = 29) rapid sequence induction and endotracheal intubation, in 2% (*n* = 14) cardiopulmonary resuscitation, and in 0.4% (*n* = 3) a chest tube insertion had to be performed onsite prior to HEC extraction. The most common medical intervention onsite is analgesia or analgosedation, in 17% (*n* = 142) fentanyl or ketamine was used in doses of ≥ 0.2 mg or ≥ 50 mg, respectively.

**Conclusions:**

As these interventions have to be performed in challenging terrain, with reduced personnel resources, and limited monitoring, our results point out the need for physicians onsite who are clinically experienced in these procedures and specially and intensively trained for the specific characteristics and challenges of HEC rescue missions.

## Introduction

Survival of severely injured patients is time dependent. It is known that the use of a helicopter emergency medical service (HEMS) can significantly shorten rescue times, especially in mountainous areas [1], and can improve patient outcomes [2]. Due to the difficult terrain in the mountains, landing a helicopter is not always possible, and hovering and human external cargo (HEC) operations such as helicopter hoist operations (HHO) or longline/human cargo sling (HCS) operations are utilized [3].

Besides reaching patients in difficult terrain and improving transport times, HEC-equipped HEMS can deliver highly trained medical providers to the scene, allowing the patient to receive time-critical medical interventions. However, this benefit of advanced medical care at the accident site must be balanced against the increased risk of incidents and fatalities for emergency medical care providers [4, 5]. Furthermore, ensuring that the HEC skills of all providers remain current is also associated with a considerable financial and training burden. The question arises as to whether having advanced medical care providers onsite is mandatory. To date, few data [6, 7] have been published investigating the epidemiology of and need for advanced medical interventions under the difficult environmental and logistical conditions of mountain HEC rescue missions.

Air Zermatt HEMS is a commercial rescue service founded in 1968 and based in Zermatt, Switzerland. It covers an area of 2621 km^2^ with a resident population of about 82,700 plus a high seasonal tourist peak, with greater than 2 million hotel guests a year. Two twin helicopters (Bell 429) capable of HEC provide 24-h HEMS service. Around 1700 rescue missions are performed each year, about 93% of which are primary missions. Ranging from road traffic accidents to mountain accidents at high altitude (> 4000 m above sea level). On average, 265 missions per year are HEC extrication, either by HHO or HCS. The HEMS crew includes a pilot, a certified flight paramedic with an additional aeronautical and winch operator training, and a physician. The physicians are consultants in anesthesia and/or intensive care medicine who work part-time as HEMS crew members for Air Zermatt and part-time in the hospital. The physicians have completed an additional training course in mountain emergency medicine as recommended by the International Commission for Alpine Rescue (ICAR). Because the paramedics serve as winch operators, they are not available for patient care on-site in case of an HEC rescue.

If the operation site is expected to be in challenging or exposed terrain (risk of falling, risk of avalanches, sheer rock faces, steep glaciers, etc.), a certified mountain guide with additional training as a technical rescue specialist (and medical training in basic life support) joins the team. In this case the HEMS crew first assesses the terrain and related risks as well as the patient’s condition while flying over the accident site, then decides whether to deposit the mountain guide and physician together (usually via a hoist operation or hovering) or to set down the mountain guide alone to evaluate the situation and to decide whether safety precautions are necessary prior to medical care.

The aim of this study was to investigate characteristics of mountain rescue missions with HEC, describing the type, frequency and timing of medical interventions performed in relation to the severity of injuries. The study results can help to optimize the use of educational, human and logistical resources and implement operative protocols.

## Methods

We retrospectively reviewed all rescue missions conducted by Air Zermatt from January 2010 to September 2016. Using the 7-level scale of the National Advisory Committee for Aeronautics (NACA), the severity of the injury or illness was graded by the physician immediately after the rescue mission, based on the mechanism of injury and clinical parameters [2] . The medical reports of all HEC rescue missions involving patients with a NACA score ≥ 3 [8, 9] were independently analyzed by three authors (UP, DA, JK). Data extrication was based on the Utstein Style and the International Alpine Trauma Registry [9, 10]. These data included the patient’s injury pattern, accident and mission characteristics, time at the scene and medical interventions (Table 1) provided before and after HEC extrication.

**Table 1:** List of medical interventions requiring a physician onsite

● High-dose pain medication (fentanyl ≥0.2 mg, ketamine ≥50 mg)	
● Vasoactive drugs (adrenaline, noradrenaline, ephedrine, phenylephrine)	
● Any other intravenous medication	
● Cardiopulmonary resuscitation (CPR)	
● Rapid sequence induction (RSI) of anesthesia and advanced airway management	
● Reduction of dislocated joints/fractures (with signs of neurological/vascular impairment)	
● Insertion of a chest drain	

Data were analyzed using SPSS statistics software (IBM Analytics, New York, USA). Data are presented as absolute and relative numbers, median with interquartile range (IQR) and range or mean as appropriate. Categorical and continuous data were compared using Fisher’s exact test. A *p*-value < 0.05 was deemed statistically significant.

## Results

**Fig. 1:**
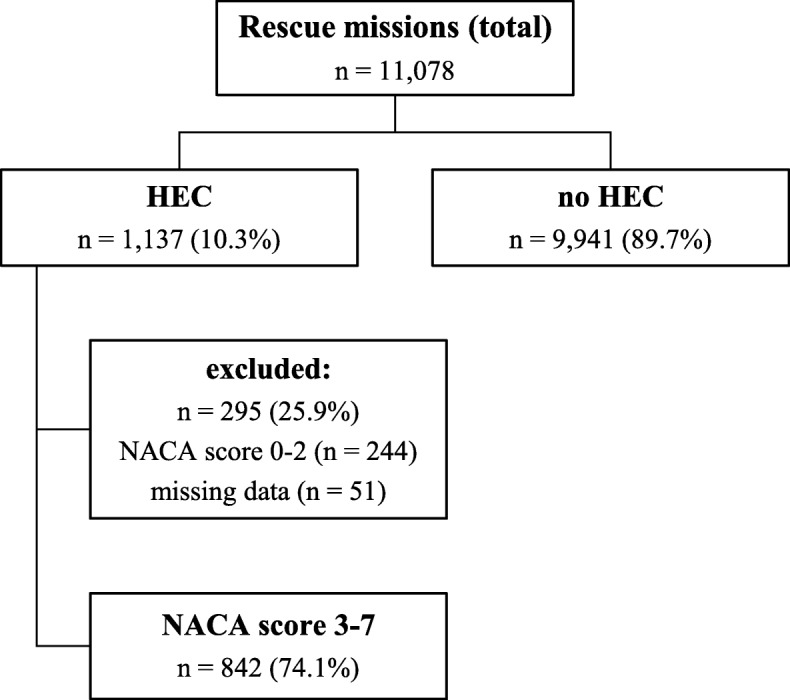
HEMS rescue missions between January 2010 and September 2016 and patient selection. *HEC* human external cargo mission, *NACA* National Advisory Committee for Aeronautic

Eleven thousand seventy-eight rescue missions were conducted between January 2010 and September 2016. Of these missions, 1137 (10%) required a HEC rescue (Fig. 1).

After exclusion of operation protocols with missing data (*n* = 51) or NACA scores of 0–2 (*n* = 244), 842 (74%) protocols were included in the study. There were 551 (65%) male and 291 (35%) female patients with a mean age of 44 years (IQR 28–59). Sixty patients (7%) were < 15 years old and 85 (10%) were older than 70.

### Accident and mission characteristics

Most of the victims requiring HEC rescue were injured while practicing winter sports or recreational activities in the mountains during the summer (hiking, mountaineering, climbing, etc.). A list of the activities performed at the time of injury is presented in Table 2.

**Table 2:** Activity at the time of injury or medical emergency (*n* = 842)

Activity	n (%)
Downhill skiing	275 (33)
Hiking	149 (18)
Mountaineering	123 (15)
Ski touring	41 (5)
Snowboarding	39 (5)
Working	34 (4)
Mountain biking	32 (4)
Paragliding	15 (2)
Motor biking	9 (1)
Off-piste skiing	9 (1)
Sledding	8 (1)
Other^a^	55 (7)
Unknown	53 (6)

^a^e.g. car driving (*n* = 4), pedestrian (*n* = 1), airplane (*n* = 1), ice-climbing (*n* = 1)

The majority of trauma events were ski-related accidents (*n* = 275, 33%), followed by hiking (*n* = 149, 18%). Forty-one (4%) accidents were reported on easily accessible terrain (home, road), 316 (37%) on difficult terrain (ski slopes, forest), and 437 (52%) in extremely difficult alpine terrain such as high in the mountains. Data on terrain were missing for 53 (6%).

The median time from emergency call to HEMS take-off for HEC rescue missions was 5 min (IQR 4–8 min; range 0–934 min), and the overall median on-scene time in a HEC mission was 26 min (IQR 20–26, range 1–211 min). Table 3 shows on-scene times in relation to NACA scores.

**Table 3:** Total on-scene time in relation to NACA score

NACA	Minimum [min]	Maximum [min]	Mean [min]	Median [min]	Total, n
3	1	155	20	23	588
4	1	211	34	32	144
5	1	129	45	39	54
6	41	75	58	60	6
7	1	183	59	53	50

*NACA* National Advisory Committee for Aeronautics

Of the victims with time-critical and possibly life-threatening injuries (NACA ≥4), 33% reached the hospital within 60 min, 35% in 61–90 min and 32% more than 90 min after alarm.

### Injury pattern

In patients requiring HEC extrication, trauma to the lower extremities (*n* = 391, 38%) and upper extremities (*n* = 250, 19%) accounted for 641 (57%) of all injuries, followed by 188 (16%) head injuries and 113 (11%) spinal trauma. In 566 (67%) cases a single diagnosis was made, in 102 (12%) two, and in 46 (5%) three or more suspected diagnoses. The incidence of suspected injury patterns is presented in Table 4.

**Table 4:** Incidence of injuries over body regions (some patients injured more than one body region)

Body region	n (%)
Lower extremities	391 (38)
Upper extremities	250 (19)
Head	188 (16)
Spine	113 (11)
Chest	82 (8)
Pelvis	61 (6)
Abdomen	20 (2)

### Severity of injuries and illnesses

The total number of medical (i.e., not trauma-related) illnesses in HEC rescue missions was much smaller (*n* = 89, 11%), but those patients presented with a significantly higher NACA score when compared to trauma victims (*n* = 753, 89%) (*p* < 0.001).

Overall, 50 (6%) of the victims in HEC rescue missions were declared dead upon arrival of HEMS or died during prehospital treatment (NACA 7) (Table 5).

**Table 5:** Distribution of NACA score in trauma and non-trauma victims

NACA	Trauma, n (%)	Non-trauma, n (%)	total, n (%)
3	548 (65)	40 (5)	588 (70)
4	120 (14)	24 (3)	144 (17)
5	43 (5)	11 (1)	54 (6)
6	1 (0.2)	5 (0.8)	6 (1)
7	41 (5)	9 (1)	50 (6)
total	753 (89)	89 (11)	842 (100)

In 254 (30%) cases, patients presented in a possibly life-threatening condition and were triaged with a NACA score between 4 and 6.

### Medical procedures provided before and after HEC extrication

The majority of the interventions (*n* = 775, 92%) in HEC rescue missions were performed onsite before HEC evacuation (Table 6).

**Table 6:** Medical interventions before and after human external cargo (HEC) extrication

Medical intervention	before HEC, n (%)	after HEC, n (%)
i.v. line	573 (68)	4 (1)
Analgesia (low dose)^a^	430 (51)	47 (6)
Analgesia (high dose)	142 (17)	20 (2)
Drugs (except analgesia and vasoactive drugs)	101 (12)	27 (1)
Volume resuscitation ≥1000 ml	39 (5)	3 (0.4)
Endotracheal intubation/RSI (excluding CPR patients)	29 (3)	14 (2)
CPR	14 (2)	2 (0.2)
Vasoactive drugs^b^ (excluding CPR patients)	19 (2)	6 (1)
Ventilation (bag-mask ventilation) (excluding CPR patients and patients requiring RSI)	20 (2)	5 (1)
Joint reduction (with signs of neurological/vascular impairment)	12 (1)	0
Chest tube	3 (0.4)	0

^a^Low-dose analgesia is defined as fentanyl < 0.2 mg or ketamine < 50 mg

^b^Adrenaline, noradrenaline, ephedrine, phenylephrine

*CPR* cardiopulmonary resuscitation, *HEC* human external cargo extraction, *RSI* rapid sequence induction

Administration of analgesia was the most common intervention: 68% (*n* = 572) of all rescued patients received analgesia before HEC extrication and 8% (*n* = 67) received analgesia afterward. Fentanyl was the most commonly used analgesic drug (*n* = 507, 89%), followed by ketamine (*n* = 140, 24%). In 75 patients (13%), a combination of both analgesics was used. Volume resuscitation of ≥1000 ml had to be performed on-site and prior to HEC extrication in 5% (*n* = 39) of the patients. Endotracheal intubation was performed before HEC extrication in 29 (3%) patients and afterward in 14 (2%). In three patients (0.4%) a chest tube had to be placed prior to HEC extrication.

## Discussion

Our data show that in alpine air rescue, HEC missions occur frequently, and numerous advanced medical interventions need to be performed onsite in challenging terrain. These rescue situations are not comparable to the in-hospital or the usual pre-hospital conditions in ambulances, patients’ domiciles, etc. Therefore, the medical team onsite has to be highly trained in medical skills as well as non-medical skills such as mountaineering, safety management, etc.

### Medical interventions

In our study, we found that the majority of medical interventions are performed at the site of the accident prior to HEC extrication. In 66 patients (8%) emergency medical interventions (cardiopulmonary resuscitation, ventilation, rapid sequence induction, endotracheal intubation, chest tube insertion) had to be performed urgently onsite due to an immediately life-threatening condition such as cardiac arrest, acute respiratory failure, cardiocirculatory collapse, or pneumothorax. In 638 patients (76%), medical interventions were indicated not due to direct life-threatening conditions but rather to signs of neurological/vascular impairment in injuries to the extremities as well as to improve patients’ comfort during the HEC rescue maneuver. 142 patients (17%) needed high-dose analgesia to ensure an extrication with as little pain as possible.

First, this emphasizes that the medical team taking part in HEC mountain rescue missions must be able to perform the entire spectrum of life-saving emergency procedures under often extremely difficult environmental conditions and with limited personnel. Second, the medical team needs extensive experience in safe and efficient analgesia and analgosedation, as there is very limited monitoring and no possibility to perform medical interventions during HEC extrication (for example bag-mask ventilation in case of respiratory arrest). Usually, in the European setting of prehospital emergency medicine, only physicians with extensive clinical experience have the training needed to perform these invasive procedures safely under these difficult environmental conditions. Therefore, our results also highlight the need for physicians onsite in a considerable number of HEC missions. In other emergency medical service systems deploying paramedics onsite with extensive in-hospital experience, a different picture can emerge.

### Airway/breathing

In European EMS, endotracheal intubation is currently considered the gold standard for advanced pre-hospital airway management [11]. Whereas Gries et al. reported endotracheal intubation in 13.5% of HEMS rescue missions and 4.4% of ground-based rescue services [12], we found that endotracheal intubation is required less frequently (*n* = 43, 5%) in our collective of patients requiring HEC extrication. Similarly, chest tube insertions are performed only rarely during ground-based EMS missions (0.08%) but in 1.1% of all HEMS rescue missions. In our collective of HEC rescue patients, chest tubes had to be inserted in 0.4% of all patients already prior to extrication and thus considerably more frequently than in ground-based EMS but less frequently than in the entirety of HEMS rescue missions. This might be explained by the fact that, due to logistics and long transportation times in the alpine terrain, a HEMS team is dispatched more frequently to less severely injured patients than in non-alpine areas.

### Analgesia

Besides stabilization of vital functions, pain therapy is one of the fundamental tasks in prehospital care. However, in emergency situations patients frequently do not receive any pain treatment at all or, despite high pain intensity, treatment remains insufficient [13]. There are various reasons for inadequate prehospital analgesia, but fear of side effects and underestimation of the duration of emergency care are the most frequently stated ones [5]. Our results show that in more than three quarters of the patients, pain medication was administered by the physician onsite. Sufficient analgesia under limited patient monitoring can ensure a rapid rescue and evacuation from technically demanding terrain, and is thus an important contribution of the physician onsite to the success and safety of the rescue mission. Moreover, even patients in harsh environmental conditions have the right to sufficient analgesia during HEC evacuation.

### Timing

When discussing the need for physicians onsite, the issue of prolonged pre-hospital time is frequently raised. It is widely accepted that early admission of a severely injured patient to a trauma center within the golden hour of shock is associated with reduced mortality, and 60 min is the upper limit acceptable for total prehospital time, as recommended in trauma management guidelines [14, 15]. However, our results in mountain rescue HEC missions show a prolonged total pre-hospital time – exceeding 60 min – in 67% of patients. This is in accordance with recent data from the International Alpine Trauma Registry (IATR) showing that mountain-rescue operations have longer total out-of-hospital times (mean 117.4 ± 142.9 min standard deviation) than rescue operations in non-alpine areas (68.7 ± 28.6 min) (10, 16).

There are several factors potentially contributing to longer treatment-free intervals for alpine rescue (mean 59.1 ± 139.5 min. SD) vs. urban/suburban rescue missions (19.7 ± 142.9 min. SD). These include the necessity for complex HEC rescue maneuvers to gain access to the patient, bad weather conditions, and long flights from remote alpine areas to level 1 trauma centers (30–40 min in the operational area of Air Zermatt HEMS). Medical interventions performed on the scene and not delayed until hospital arrival might be even more important in rescue missions with prolonged out of-hospital times and in particular long transportation times than in urban rescue, the more so as interventions during air transport are usually impossible due to limited access to the patient. That prolonged pre-hospital times are not necessarily harmful, is supported by IATR data showing that in-hospital mortality in trauma patients transported from alpine regions is comparable to that of patients from urban areas, despite longer pre-hospital times and higher injury severity scores [16]. Furthermore, overall time from the accident until the end of emergency department (ED) treatment is equal for severely injured patients undergoing e.g. endotracheal intubation and pleural decompression, regardless of the point in time (pre-hospital or in-hospital) that these interventions are performed [17].

### Education

Our data show that invasive and demanding emergency medical procedures such as rapid sequence induction (RSI) or chest tube insertion are performed less frequently during HEC rescue missions than during HEMS missions in the non-alpine area, but much more frequently than in ground-based EMS systems. In comparison to typical HEMS rescue missions, where invasive procedures are often performed inside an ambulance or indoors, for HEC rescue missions environmental conditions are usually poor (steep terrain, snow, etc.) and personnel resources are very limited. The HEMS paramedic often serves as the winch operator and is therefore not available to treat the patient on-site. This demonstrates the need for intensive and comprehensive education and training. To perform complex and time-critical interventions (such as RSI or chest drain insertion), the necessary procedural and manual skills have to be trained and perfectly mastered in in-hospital settings (intensive care unit, emergency department, etc.) as well as out-of-hospital under emergency conditions (in ground-based EMS and non-alpine HEMS) before a physician can be signed off for HEMS rescue missions with HEC.

More than half of the HEC missions in our retrospective study were performed in steep or high alpine terrain. This underlines the need for competence in dealing with alpine dangers such as rock falls, weather changes, and avalanches. Therefore, the medical team performing mountain rescue missions has to be trained in basic mountaineering techniques such as rock climbing, ice climbing, and self-belaying. According to the Mountain Emergency Medicine Curriculum of the Swiss Society of Mountain Medicine, medical crew members in mountain rescue missions must be able to walk and climb with crampons and ice axes in easy terrain, lead climbs with mountain boots up to the difficulty level of 3 (UIAA), belay a companion for lead climbing, rapell, set up a belay (in snow, ice and rock), self-rescue out of crevasses, ski safely off piste, and search for and find avalanche victims using an avalanche transceiver [18].

To date there are no standardized guidelines or training programs for advanced life support (ALS) in the mountain rescue environment. However, there are some related courses which can provide helpful training opportunities, such as the rescue modules of the Diploma in Mountain Medicine and the Diploma in Mountain Emergency Medicine (provided by the International Climbing and Mountaineering Federation, International Commission for Alpine Rescue and International Society of Mountain Medicine) or the Advanced Wilderness Life Support® course (provided by the Wilderness Medical Society) [10].

If invasive interventions are unavoidable before HEC extrication, a careful risk/benefit analysis should be performed, following clear and predefined standard operating procedures (SOP) [19]. Pit stop-like training should be drilled periodically. We encourage these organizations to establish continuous and inter-professional simulation training [20, 21], with a strong emphasis on the safety of any ALS interventions, as well as maneuvers for the HEC extrication of the injured patient. Patient and team member safety in HEC operations is a very important issue. Victims and rescuers can face extreme and dangerous environments. Medical tactics must consider those factors, and a thorough risk-benefit analysis of medical interventions onsite must be done. When a mountain guide joins the HEMS team for a HEC rescue mission the crew members have to organize themselves in an “ad hoc” fashion and within minutes after the operation has already commenced [19]. This highlights the need for joint and structured training that not only includes technical skills, but also non-technical skills, such as safety briefing, structured communication, situational awareness, decision making, as well as stress and resource management.

Further development of appropriate protocols for the medical team could help make onsite decisions clearer and easier in the future. Additionally, continuous adoption of the strategy of HEC rescue missions to changes in weather conditions and unexpected technical difficulties has to be trained (in simulator-based scenarios for example).

### Limitations

Our study has some limitations. Firstly, this is a single-center study. Our rescue characteristics are very specific and selective due to the operational area of Air Zermatt, famous as a vacation destination with focus on skiing and mountaineering. Second, the quality and completeness of some data reported may have suffered from the retrospective design of our study. Third, we were unable to validate the pre-hospital diagnosis made by the HEMS team or to determine in-hospital outcome because of the lack of related hospital follow-up in our database. Fourth, validity of the NACA score is limited as it seems to be partly dependent on subjective factors [22, 23]. But in the prehospital setting, with the limited number of objectively measured parameters, every scoring system has its limitations. Therefore, we used the NACA score as the best available and most commonly used scoring system in prehospital emergency medicine. Finally, validity of our discussion or conclusions is partly limited by national differences in the composition of the HEMS crew and legal aspects.

## Conclusions

Invasive, life-saving procedures are frequently necessary onsite prior to HEC extrication, and often require safe analgesia/analgosedation under difficult external conditions and terrain. Besides extensive in-hospital experience in the necessary manual and clinical skills for the physician, intensive and specialized crew training is needed for the whole medical team performing HEC missions and providing HEMS service in alpine regions. Experience in working under extreme environmental conditions with reduced human resources is essential, and basic mountaineering skills have to be regularly trained.

## Abbreviations

ALS: Advanced Life Support
HCS: Human cargo sling
HEC: Human external cargo
HEMS: Helicopter emergency medical service
HHO: Helicopter hoist operation
IQR: Interquartile range
NACA: National Advisory Committee for Aeronautics
RSI: Rapid sequence induction
SOP: Standard operating procedure
